# Novel CSF biomarkers for diagnosis and integrated analysis of neuropsychiatric systemic lupus erythematosus: based on antibody profiling

**DOI:** 10.1186/s13075-023-03146-z

**Published:** 2023-09-08

**Authors:** Jiali Ni, Chen Chen, Shuangan Wang, Xuan Liu, Liping Tan, Li Lu, Yu Fan, Yayi Hou, Huan Dou, Jun Liang

**Affiliations:** 1 Department of Rheumatology and Immunology, Nanjing Drum Tower Hospital, The Affiliated Hospital of Nanjing University Medical School, Nanjing, 210008 China; 2 The State Key Laboratory of Pharmaceutical Biotechnology, Division of Immunology, Medical School, Nanjing University, Nanjing, 210093 China; 3 Department of Clinical Nutrition, Nanjing Drum Tower Hospital, The Affiliated Hospital of Nanjing University Medical School, Nanjing, 210008 China; 4 Jiangsu Key Laboratory of Molecular Medicine, Nanjing, 210093 China

**Keywords:** NPSLE, Biomarkers, Diagnosis, Protein array, Machine learning

## Abstract

**Background:**

Neuropsychiatric systemic lupus erythematosus (NPSLE), with various morbidities and multiple manifestations in the central nervous system, remains a limited standard for diagnosis. Our study was to discover novel biomarkers for improving the diagnostic efficiency for NPSLE.

**Methods:**

We performed a quantitative planar protein antibody microarray to screen 1000 proteins in cerebrospinal fluid from controls, systemic lupus erythematosus (SLE, non-NPSLE) patients, and NPSLE patients. Differentially expressed proteins (DEPs) as candidate biomarkers were developed into a custom multiplexed protein antibody array for further validation in an independent larger cohort. Subsequently, we used least absolute shrinkage and selection operator regression (LASSO) analysis and multivariable logistic regression analysis for optimizing feature selection and constructing a diagnostic model. A receiver operating characteristic curve (ROC) was generated to assess the effectiveness of the models.

**Results:**

The expression of 29 proteins in CSF was significantly altered in the comparison of the three groups. We selected 17 proteins as candidate biomarkers in accordance with protein interaction analysis. In the larger cohort, we identified 5 DEPs as biomarkers for NPSLE, including TCN2, CST6, KLK5, L-selectin, and Trappin-2. The diagnostic model included 3 hub proteins (CST6, TCN2, KLK5) and was best at discriminating NPSLE from SLE patients. These CSF biomarkers were also highly associated with disease activity. In addition, there were 6 molecules with remarkable changes in NPSLE CSF and hippocampus, which indicated the consistency of the environment in the brain and the promising molecular targets in the pathogenesis of NPSLE.

**Conclusions:**

The dual-chips screening strategy demonstrated KLK5, L-selectin, Trappin-2, TCN2, and CST6 as CSF biomarkers for diagnosing NPSLE.

**Supplementary Information:**

The online version contains supplementary material available at 10.1186/s13075-023-03146-z.

## Background

As the second leading cause of mortality in systemic lupus erythematosus (SLE) patients, neuropsychiatric systemic lupus erythematosus (NPSLE) has attracted considerable attention since the American College of Rheumatology (ACR) unveiled canonical nomenclature and classification criteria for NPSLE in 1999 [1, 2]. The 19 NPSLE symptoms comprise 7 focal neurological symptoms and 12 diffuse central nervous system (CNS) symptoms [3]. Diagnostic approaches have also been enriched constantly along with massive disease symptoms, including serological and CSF testing, neuroimaging, and neuropsychological testing [[4, 5]. However, ACR criteria have not been shown to correlate with clinical diagnosis. For example, the poor correlation between nonspecific changes in brain structure and the incidence of NPSLE has led to the fact that the results of neuroimaging were often dependent on the judgment of experienced physicians [6]. There was still a lack of specific non-invasive diagnostic biomarkers, leading to the variable prevalence of NPSLE in SLE patients from diverse studies, estimated between 12 and 95% [7–9]]. The difficulty of catching the disease early caused the diagnosis of NPSLE patients which have been often confirmed during the active phase of the disease, when irreversible damage to brain tissue has occurred.

Cerebrospinal fluid (CSF) is a colorless and transparent liquid filled with the ventricles, subarachnoid space, and central canal of the spinal cord, which is involved in the immune regulation, metabolism, supply of nutrients, and elimination of metabolic waste in the brain [10]. Indeed, it has been reported that NPSLE patients showed nonspecific abnormalities, such as increased white blood cell count (WBC) and elevated albumin in CSF [11]. When infection, inflammation, tumor, edema, and obstruction occur in CNS in NPSLE patients, they need further detection of specific markers in CSF to directly reflect the pathological changes in the brain, which assist in the diagnosis of NPSLE [12]. In brief, we looked forward to dig out novel biomarkers in CSF from NPSLE patients and discover a new strategy for finding diagnostic indicators of NPSLE.

Our study focused on the antibodies-based microarrays to dig out DEPs as candidate biomarkers in CSF for NPSLE and hoped that these technology benefits to identify easily overlooked low-abundance proteins. The glass-slide-based protein array was designed and fabricated to screen and quantify 1000 proteins in CSF from NPSLE, SLE patients, and controls. We next customized a multiple proteins-multiplexed antibody microarray to evaluate the performance of differentially expressed proteins in a larger and independent cohort. Further analysis with clinical and pathological indices has authenticated the diagnostic model (including CST6, TCN2, and KLK5) as the best discrimination of NPSLE from SLE, which has the potential to assist the clinical development of a novel diagnostic system.

## Methods

### Patients and clinical sample collection

Patients were recruited from the Department of Rheumatology, Nanjing Drum Tower Hospital, Nanjing, China. They were all diagnosed according to the ACR criteria. Patients underwent the necessary tests to determine disease activity by calculating the Systemic Lupus Erythematosus Disease Activity Index (SLEDAI more than 6 represented an active phase of the disease). NPSLE patients were defined as exhibiting ≥1 neuropsychiatric disorder within 2 weeks before inclusion. Criteria for exclusion included patients with previous and/or known substance abuse, alcoholism, diabetes, stroke and/or renal insufficiency, complex lupus dermatosis manifested by rash and/or lesions, Systemic sclerosis, myositis, other autoimmune diseases, cancer, infections, or patients who have received glucocorticoids or immunosuppressive agents in the past 6 months.

Participants completed a standardized medical history and laboratory analysis and gave informed consent to all studies. We established experimental protocols according to the guidelines of the Declaration of Helsinki. All the studies were approved by the ethics committee at the Affiliated Drum Tower Hospital of Nanjing University Medical School (approval number: 2022-563-02). The primary cohort for initial screening comprised 2 controls, 3 patients with SLE, and 3 patients with NPSLE (Table S1). The independent cohort with expanded size for further validation comprised 9 controls, 18 patients with SLE, and 37 patients with NPSLE (Table S2).

Clinical staff followed standard aseptic procedures after administering local or general anesthesia to the patient. The waist of the patient was punctured to collect CSF, and the plan of collecting CSF from the medulla oblongata region of the cerebellum was an alternative. CSF samples were immediately aliquoted into siliconized polypropylene tubes and immediately frozen on dry ice. All samples were stored at −80°C or in liquid nitrogen prior to measurement.

### One thousand-plexed proteins array and 17-plexed customized proteins array screening

The 1000 proteins array was a combination of 25 non-overlapping glass slide-based antibody arrays (GSH-CAA-X00, RayBiotech, Norcross, GA, USA). The 1000 proteins and raw data are listed in Table S3. After 2h incubation with the CSF samples, the target proteins were captured by the antibodies printed on the solid surface. A second biotin-labeled detection antibody was added to incubate 2h, which recognizes a different epitope of the target proteins. The protein-antibody-biotin complex could then be visualized through the addition of the streptavidin-conjugated Cy3 equivalent dye for 2h. The array utilized a highly sensitive and stable fluorescent readout which can be detected by the laser fluorescent scanner (InnoScan 300 Microarray Scanner, Innopsys, France). The raw data were extracted by GenePix and subtracted from the median background signals, normalized to the positive control by the Analysis Tool software (GSH-CAA-X00-SW, RayBiotech). Comparison of signal intensities for antigen-specific antibody spots between and among array images determined relative differences in expression levels of each protein for further analysis.

After the first screening of 1000 proteins and investigation of literature, 17 proteins as candidate biomarkers were selected and assembled into a customized protein array for further validation (CUSTOM-AAH-17, RayBiotech). The 17 proteins and raw data are listed in Table S4. The operating principle of the 17-plexed proteins array was roughly similar to the 1000-plexed proteins array.

### DEP analysis, enrichment analysis, and network visualization

The normalized data were analyzed by the moderated t-statistics. DEPs were defined as those with adjusted *P* value (corrected by Benjamini–Hochberg test [13]) less than 0.05, and foldchange (FC) over 1.2 or less than 0.83 (absolute logFC > 0.263), which were presented as dot plots.

Enrichment analysis of Gene Ontology (GO) was finished by the “clusterProfiler” package from R/Bioconductor, using Fisher’s exact test to determine whether there exists more overlapping in the gene list and the GO annotation list. The criteria for selection were that the number of DEPs falling on a term was ≥2, adjusted *P* value <0.05. The enrichment score generated by -log10 (adjust P value) implied the importance of the pathway.

We uploaded 29 proteins into the STRING database, choosing medium confidence (>0.4) to construct protein–protein interaction (PPI) network. Cytoscape 3.9.1 were devoted to visualizing the PPI network and selecting proteins for further validation by calculating the value of betweenness centrality (BC). Similarly, we used the comprehensive Cytohubba plugin to distinguish DEPs from the PPI network through the method of maximal clique centrality (MCC) [14].

### Construction and evaluation of the diagnostic model

The least absolute shrinkage and selection operator (LASSO) algorithm was used to identify optimal proteins with higher diagnostic values [15]. We selected hub proteins with P values < 0.05 as the final parameters of the diagnostic model. And, multivariable logistic regression analysis was applied to construct the diagnostic model by using hub proteins screened from LASSO regression. The features of the model included an odds ratio (OR) having a 95% confidence interval (CI) and as *P* value. We applied R software and calculated the area under the curve (AUC) of the receiver operating characteristic (ROC) to evaluate the diagnostic value of the model.

### Statistical analysis

All data were analyzed and plotted by using GraphPad Prism (version 9.0, GraphPad, San Diego, CA, USA), SPSS (version 25, Inc., IL, USA), Microsoft Excel 2019, or R software. One-way analysis of variance (ANOVA) test was taken in use to perform statistical analysis, using the least significant difference (LSD) test as a post hoc test. *P* value < 0.05 was considered statistically significant. Moreover, we established the binary logistic regression models for the individual candidate biomarkers, and next used the Spearman and Pearson methods for the correlation analysis, and calculated sensitivity, specificity, and AUC by ROC analysis.

## Results

### One thousand CSF proteins array screening

**Fig. 1:**
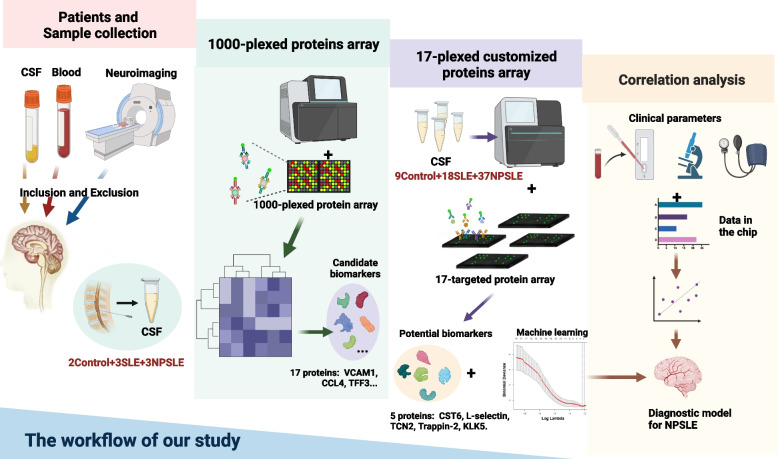
The workflow of our study. We firstly used the initial cohort and 1000-plexed proteins array to select candidate biomarkers, then used the independent cohort and 17-plexed customized proteins array to obtain 5 potential biomarkers

**Fig. 2:**
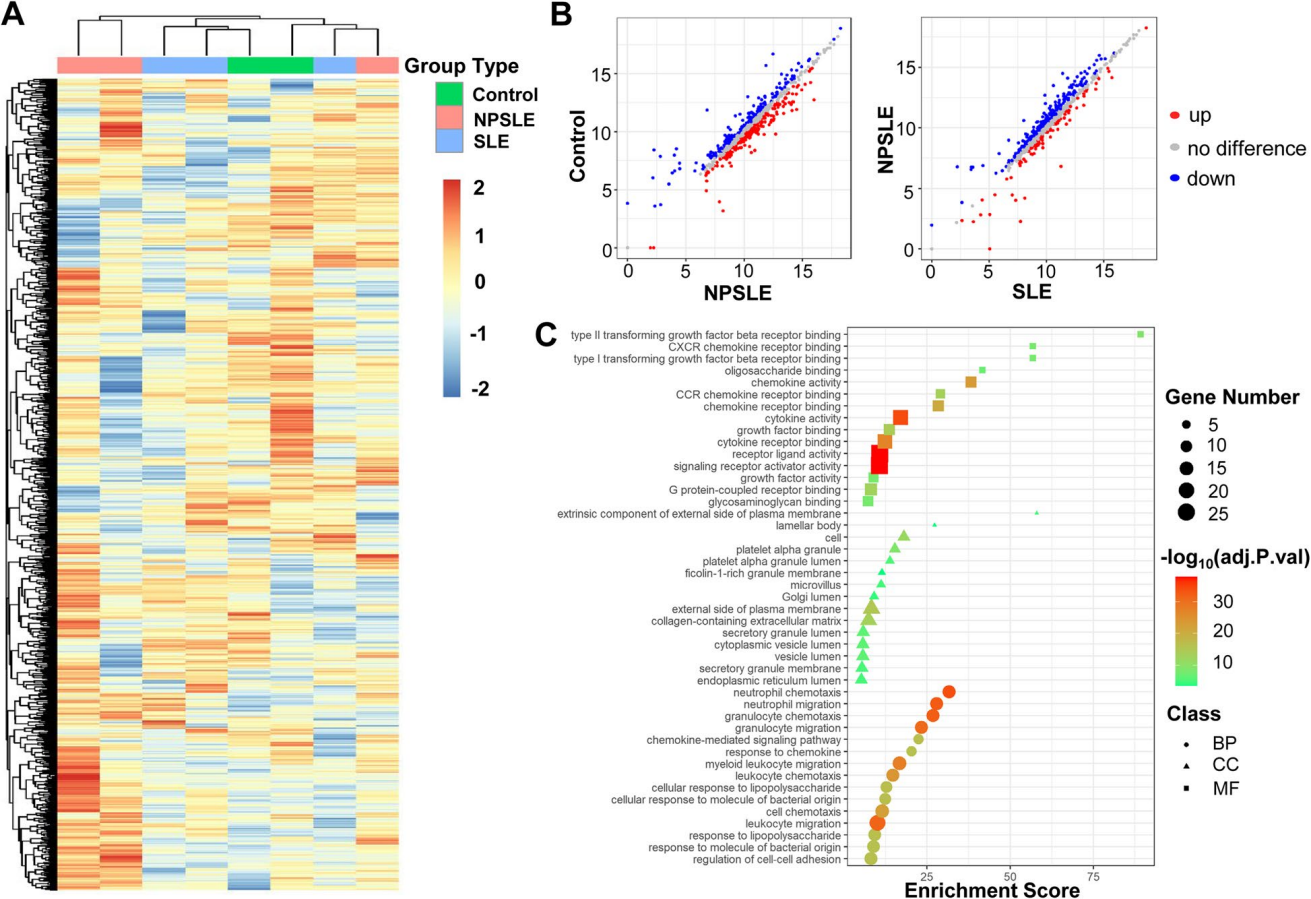
One thousand-plexed proteins array of CSF samples. **A** Heatmap of 1000 proteins array clustered by patients’ groups. Each column represented one sample, and each row represented one protein, with red indicating overexpression and blue indicating low expression, compared with the median expression for the protein. **B** Scatter plot showed the differentially expressed proteins. The axes respectively represented the average expression value of each protein from a different group, with red indicating up-regulated and blue indicating down-regulated.** C** GO enrichment analysis of DEPs between SLE and NPSLE patients including biological process, cellular component, and molecular function, arranged by enrichment score

As illustrated in Fig. 1, we firstly collected CSF samples from patients in three groups: controls, SLE, and NPSLE patients. A 1000-plexed microarray was used for the screening of 1000 human proteins in CSF by specific binding of antigen and antibody. The expression of 1000 proteins was applied to generate the heatmap among three groups, which showed DEPs in NPSLE patients compared with SLE patients and controls (Fig. 2A). In order to visualize the expression of proteins more intuitively and conveniently, we showed 256 significantly elevated proteins and 233 significantly decreased proteins in NPSLE patients compared with controls as the scatter plot (Fig. 2B). Similarly, there were 86 DEPs in NPSLE patients compared with SLE patients that comprised 21 down-regulated proteins and 65 up-regulated proteins (Fig. 2B).

There were striking differences in a series of biological processes (BPs) between SLE and NPSLE patients, focused on neutrophil chemotaxis and migration, granulocyte chemotaxis and migration, etc. (Fig. 2C). Previous studies have already discovered that the cerebrovascular inflammation, the reduction of neuronal synapses, and the activation and phagocytosis of microglia have all played important role in the function of CNS and pathogenesis of NPSLE [16, 17]. In particular, these BPs also showed significant differences in NPSLE compared with SLE patients, such as regulation of neuroinflammatory response, vasculogenesis, and central nervous system neuron axonogenesis (data not shown).

### Further screening for 17 candidate biomarkers

**Fig. 3:**
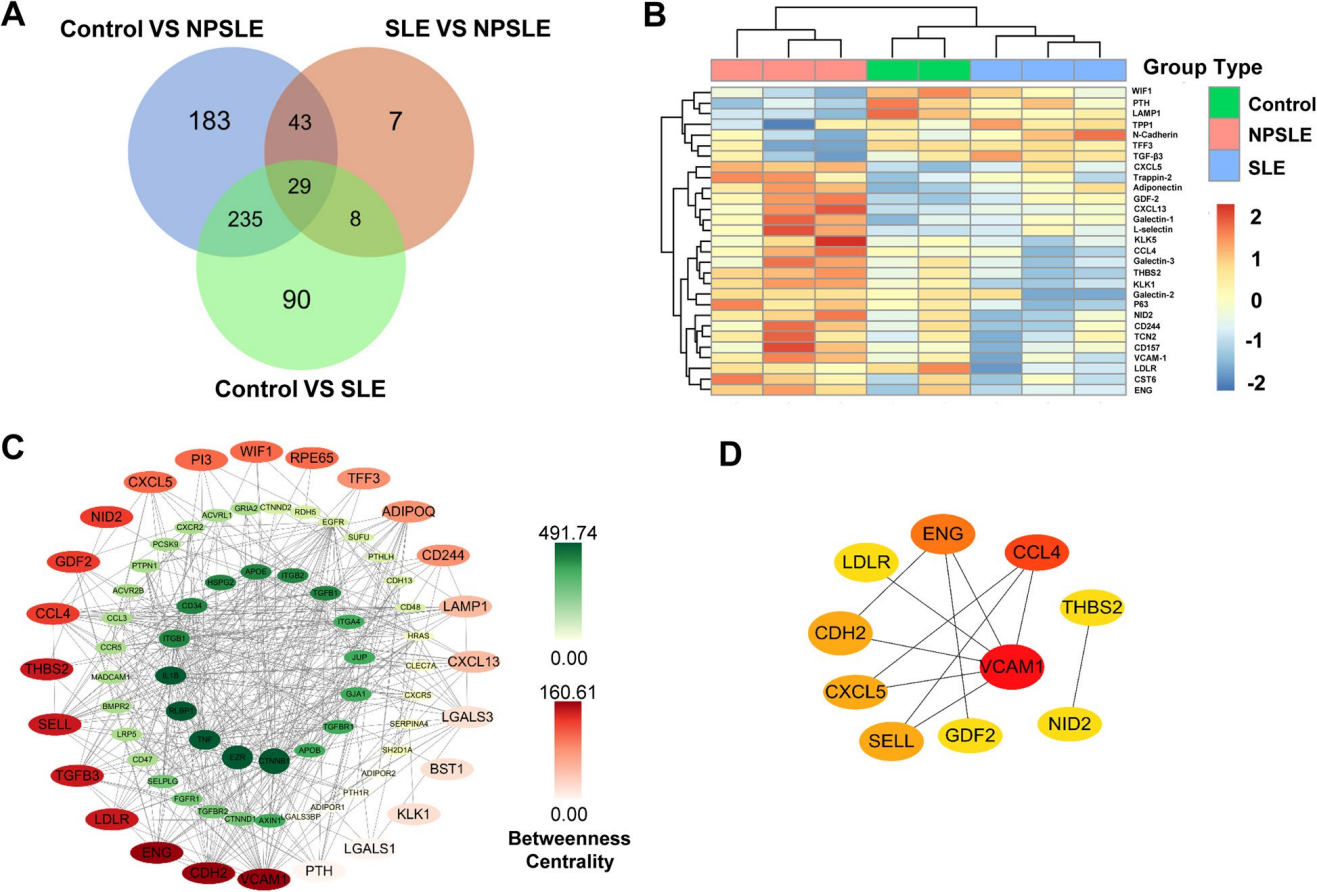
Candidate biomarkers based on the screening of 1000 proteins were selected for further validation. **A** Venn diagram depicted concordant proteins in DEPs from control VS SLE, control VS NPSLE, and SLE VS NPSLE. **B** Heatmap of 29 intersected proteins clustered by patients’ groups in the 1000-plexed proteins array. **C** PPI network of 29 proteins and derived proteins visualized by Cytoscape, with red indicating 29 proteins (no-interaction proteins were deleted) and green indicating derived proteins. **D** Key proteins were shown by the algorithm of Maximal Clique Centrality in Cytohubba. The darker the node, the higher the score

All DEPs from the pairwise comparison among three groups were intersected and shown in a Venn diagram (Fig. 3A). It is indicated that 29 DEPs were overlapped after multiple testing corrections and complied with the following phenomenon: the trends of the variably expressed proteins were consistent between NPSLE and SLE patients and between NPSLE and control groups. The mean/median and fold change of 29 DEPs in NPSLE and SLE patients were concluded in Table S5. We have also retrospected the expression of the shared common DEPs by a heatmap regarding log fold change to understand more intuitively (Fig. 3B).

There were more stringent standards for determining which candidate CSF proteins would be selected for further validation. We were firstly intrigued with 11 proteins that have never been reported to be associated with SLE or NPSLE (CDH2, WIF1, etc.). Some proteins were not chosen on account of diverse reasons as shown in Table S6, such as Kallikrein 1, belonging to the same family as Kallikrein 5. Besides, the PPI networks from the 29 DEPs were visualized in the CytoNCA plugin in Cytoscape for the analysis and assessment of protein interaction network centrality. As shown in Fig. 3C, 24 DEPs (red color) were connected with more numerous molecules (green color) in proximity to construct an abundant submodule network, based on the higher value of betweenness centrality (BC). So, we focused more on the proteins with the top values of BC, such as VCAM-1 and LDLR. In summary, we applied 17 proteins shown to further verification and particularly enumerated the protein names and their aliases we have used in our study to avoid confusion (Table S6).

The expression of 17 proteins was compared in three groups, whereas the radar charts for the relative protein expression rather than absolute values were depicted in Fig. S1A. It underscored the similar trends in the expression differences of these proteins. Next, we also scrutinized the PPI network mediated by 17 selected proteins from STRING analysis, using a minimum required interaction score of >0.4 (medium confidence) (Fig. S1B). VCAM-1, ENG, CCL4, and CXCL5 were the most interconnected nodes, while the PPI network was imported into the Cytohubba plugin in Cytoscape with the topological algorithms-maximal clique centrality (MCC). It captured the most essential proteins as the key proteins (top 10, 58.8%) in an interactome network (Fig. 3D). In conclusion, VCAM-1, CCL4, and ENG were the key molecules, which might participate in the pathological mechanisms of NPSLE and be the potential therapeutic targets.

### Seventeen proteins custom chip for further validation

We carried out the validation for 17 candidate biomarkers with a customized protein array. We used a principal component analysis (PCA) to reduce the dimensionality of data and interpret the variation of data. PC1 explained a large proportion of the variance (74.25%) and resulted in two non-overlapping clusters combined with PC2 (18.56%) between NPSLE and control groups, which clearly distinguish the two groups (Fig. S2A). The cumulative contribution rate of the two principal components was 93.17% between NPSLE and SLE patients (Fig. S2A). The PCA results showed that NPSLE patients were better distinguished from controls than from SLE patients, which suggested that the difference between NPSLE and SLE patients might be from lower variations in protein expression. It was consistent with the complexity of discriminating NPSLE patients from SLE patients in clinical diagnosis.

**Fig. 4:**
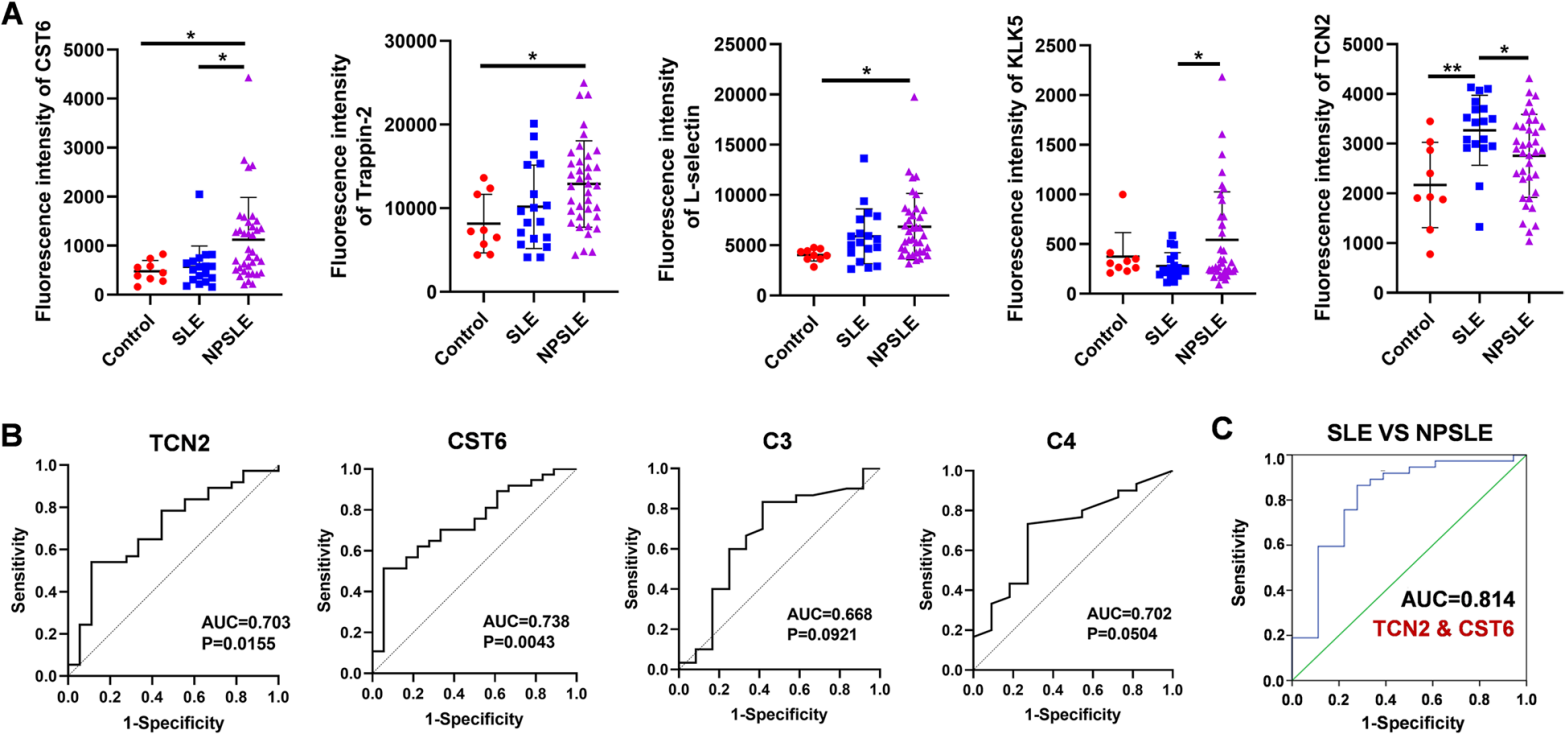
Verification of candidate biomarkers. **A** The statistical graph of fluorescence intensity of 5 proteins based on the 17-plexed customized proteins array and the data have been normalized. (*P<0.05; **P<0,01). **B** ROC curves using TCN2 (AUC=0.703, *P*=0.0155) and CST6 (AUC=0.738, *P*=0.0043), C3 (AUC=0.668, *P*=0.0921) and C4 (AUC=0.702, *P*=0.0504) for distinguish NPSLE from SLE. **C** Multiple proteins were combined into panels in distinguishing NPSLE from SLE, using logistic regression analysis, which indicated the highest diagnostic value of the combination of TCN2 and CST6 with an AUC of 0.814

There were 3 significantly elevated proteins in NPSLE patients compared with controls, including CST6, L-selectin (SELL), and Trappin-2 (PI3) (Fig. 4A). Particularly, the expression of TCN2, KLK5, and CST6 was substantially changed in NPSLE patients compared with SLE patients. Whereas the other 12 proteins showed no significant variation, high-content screening demonstrated its ability to capture low-abundance proteins precisely (such as TFF3, data not shown).

In addition to specifically detect the expression of multiple target proteins in the meanwhile, the customized array also determined the functions of candidate biomarkers by GO annotation. The top highest enrichment scores of three GO subgroups were displayed between NPSLE and control groups (Fig. S2B) or between NPSLE and SLE patients (Fig. S2C).

### Diagnostic value of biomarkers for NPSLE

To investigate whether the 5 CSF proteins as biomarkers for distinguishing NPSLE patients from SLE patients without CNS disorders, we carried out receiver operating curve (ROC) analysis in the validation cohort of NPSLE and SLE patients. Youden index showed the optimal diagnostic cut-off value of TCN2 (sensitivity: 54.05%; specificity: 88.89%) and CST6 (sensitivity: 51.35%; specificity: 94.44%), which displayed the potential to predict disease risks for NPSLE patients respectively (Table S7). The area under the ROC curve (AUC) from TCN2 and CST6 was 0.703 (*P*=0.0155) and 0.738 (*P*=0.0043) (Fig. 4B). They outperformed traditional clinical parameters for NPSLE such as C3 (AUC=0.668; *P*=0.0921) and C4 (AUC=0.702; *P*=0.0504) in serum (Fig. 4B). We next made various combinations of CSF proteins by using binary logistic regression analysis, to further improve the precision of diagnosis. The combination of TCN2 and CST6 exhibited the best diagnostic accuracy, which the AUC was 0.814 (95% confidence interval (CI): 0.682–0.945) (Fig. 4C). A similar approach was applied into distinguishing NPSLE patients from controls simultaneously. L-selectin (sensitivity: 62.16%; specificity: 72.22%), Trappin-2 (sensitivity: 89.19%; specificity: 66.67%), and CST6 (sensitivity: 51.35%; specificity: 100.00%) became an optimal combination of CSF proteins for discriminating NPSLE patients from controls (AUC=0.884; 95% CI: 0.789–0.980) (Fig. S3).

**Fig. 5:**
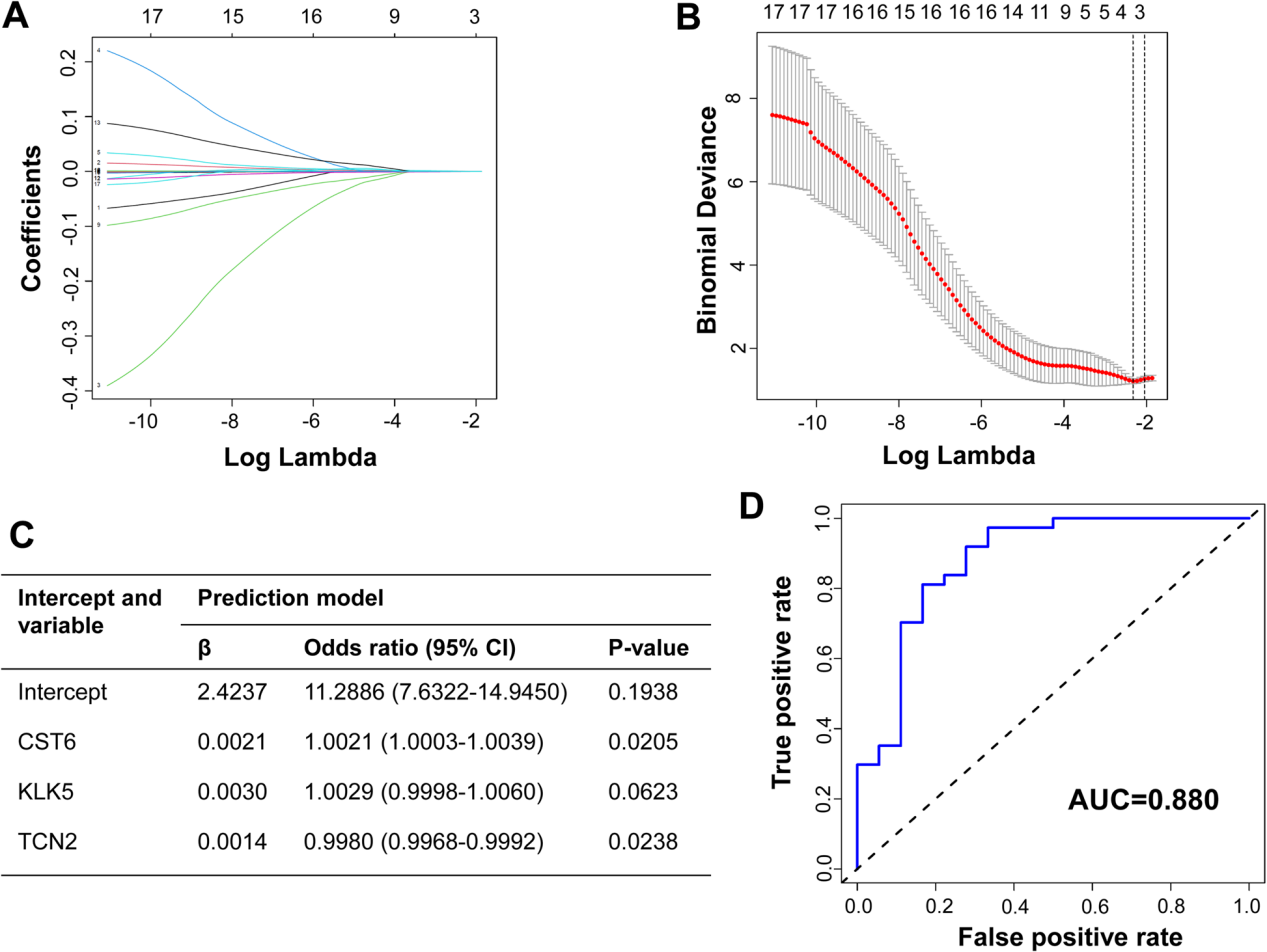
Selection of hub proteins to construct the diagnostic model between NPSLE and SLE patients. **A** LASSO regression for screening parameters and mapping each one to a curve. **B** Optimal parameter (lambda) selection in the LASSO model via minimum criteria. **C** Prediction factors from the diagnostic model for NPSLE determined by logistic regression. **D** ROC analysis of the diagnostic model

Furthermore, we also used LASSO regression algorithms to select hub proteins from 17 candidate biomarkers of NPSLE. The results showed that CST6, KLK5, and TCN2 were identified as characteristic proteins between NPSLE and SLE patients (Fig. 5A, B). We used these hub proteins to construct the diagnostic model by multivariable logistic regression analysis (Fig. 5C). Through ROC analysis, we verified that the AUC of the model was 0.880, which was higher than each individual protein (Fig. 5D). Similarly, CST6, L-selectin, TCN2, and Trappin-2 were selected to construct diagnostic model between NPSLE patients and controls (Fig. S4A–C). The AUC of the diagnostic model was 0.908 (Fig. S4D). All results exhibited the optimal diagnostic models based on CSF biomarkers for NPSLE, which indicated the importance of five hub proteins (CST6, L-selectin, Trappin-2, KLK5, TCN2) in diagnosing NPSLE.

In order to assess the correlation of 5 CSF proteins with classical clinical parameters in NPSLE, we used the Spearman correlation analysis (Table 1). According to the Spearman coefficients depicted in Table 1, three CSF proteins (CST6, L-selectin, and Trappin-2) were significantly positively correlated with immunoglobulin (IgG) in CSF. There was also a positive correlation between CST6 with Systemic Lupus Erythematosus Disease Activity Index (SLEDAI) and CST6 with albumin in CSF. It underscored the utility of CST6 in predicting various clinical metrics. TCN2 was negatively correlated with neutrophil count (NEUT) and positively associated with IgG in serum. Conversely, 5 CSF proteins exhibited poor correlation with white blood cell count (WBC) and lymphocyte (LYM) in CSF. The lumbar puncture might cause the alteration of WBC and LYM in CSF, which indicates that the cell counts should be associated with clinical manifestations to diagnose the infection of the brain. In summary, the association between CSF biomarkers and peripheral blood-related detection index was frail. The likely reason could be the natural mechanical and osmotic barriers between blood and CSF.

**Table 1:** Correlation analysis of CSF potential biomarkers versus clinical indicator

**Spearman**	**TCN2**	**CST6**	**KLK5**	**L-selectin**	**Trappin-2**
**SLEDAI**	−0.13	0.36**	−0.19	0.21	0.08
**CSF IgG**	−0.05	0.34*	−0.22	0.57****	0.36*
**CSF albumin**	−0.10	0.29*	−0.07	0.31*	0.22
**Serum IgG**	0.39**	−0.29	−0.11	−0.10	−0.25
**Serum albumin**	0.04	−0.02	−0.01	0.02	−0.02
**C3**	0.02	0.05	0.32*	0.12	0.12
**C4**	0.18	0.04	0.26	0.13	0.11
**WBC**	−0.22	−0.15	−0.13	−0.10	−0.08
**NEUT**	−0.31*	−0.17	−0.04	−0.23	−0.10
**LYM**	−0.15	−0.14	−0.13	−0.03	−0.09

Spearman analysis was used to analyze the correlation of 5 CSF proteins (TCN2, CST6, KLK5, L-selectin, Trappin-2) with clinical indexes in the independent cohort

*WBC* white blood cell count, *NEUT* neutrophil count, *LYM* lymphocyte

A *p*-value < 0.05 is considered statistically significant. *P<0.05; **P<0.01; ****P<0.0001

### Correlation of biomarkers with cognitive dysfunction in NPSLE

**Fig 6:**
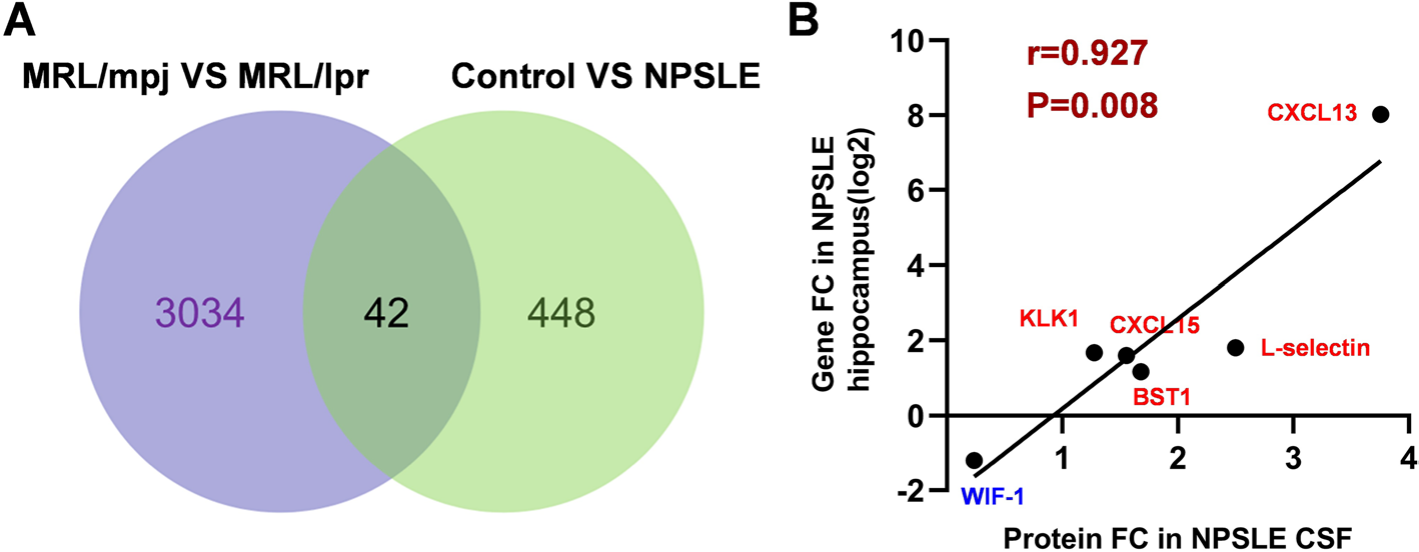
Correlation of proteins in CSF from NPSLE patients versus genes in hippocampus from MRL/lpr mice. **A** Venn diagram showed 42 common proteins in the two data subsets: DEPs between NPSLE and control groups and DEGs between MRL/mpj and MRL/lpr mice (GEO Series: GSE154288). **B** Correlation scatter diagram showed the positive relationship of the respective fold ratios of 6 candidate biomarkers from two datasheets (*r*=0.927, *P*=0.008)

To further explore the efficacy of CSF biomarkers in the diagnosis of NPSLE, we followed the applications of DEPs in cognitive dysfunction, which was one of the most typical symptoms of NPSLE. Recently, Han et al. have performed whole-transcriptome gene expression analysis of the hippocampus in MRL/lpr and MRL/mpj mice [16]. So, we searched the raw data from GEO DataSets, and intersected the subset of differentially expressed gene (|log_2_FC|>1, *P*<0.05) with the subset of DEPs from NPSLE and control groups as displayed in Fig. 6A. We identified 42 common differentially expressed molecules. More than half of the 42 molecules have been related to cognitive function on the basis of existing findings. For example, IGFBP4 involves the process of aging-related cognitive dysfunction [18]. We also needed more research to dig out the unknown relationship between the rest of proteins with cognitive function.

Twenty-nine CSF proteins were overlapped in a 1000-proteins array, which represented 29 unique molecules. The gene expression of the 29 CSF molecules was also evaluated in this database. In particular, 6 molecules showed significant differences in the hippocampus of MRL/lpr mice. As the correlation analysis indicated, there was a strong correlation between the fold ratio of 6 molecules in the hippocampus of NPSLE mice and CSF of NPSLE patients (*r*=0.927, *p*=0.008) (Fig. 6B). And it has been authenticated that most of them involved the pathogenesis of cognitive impairment, such as the expression of L-selectin was decreased with the decline of cognitive ability in Alzheimer’s disease (AD) patients [19]. In brief, we were looking forward to these proteins as biomarkers of cognitive dysfunction in NPSLE patients.

## Discussion

The pathological mechanisms of NPSLE were complicated and elusive and involved various contributing factors. Protein array as a nascent technology is critical to analyze multiple protein expressions with plentiful advantages. In our study, we have achieved a comprehensive screening of CSF 1000 proteins to discover novel biomarkers from three groups, controls, SLE (non-NPSLE) patients, and NPSLE patients. The number of overlapped DEPs was only 29, which was further whittled down to just 17. Thus, a 17-plexed specific targets array was customized to testify the diagnostic value of 17 candidate biomarkers to distinguish NPSLE patients from controls and SLE patients. Combined with machine learning algorithm, ROC analysis, and correlation analysis of clinical indicators, TCN2, CST6, Trappin-2, L-selectin, and KLK5 emerged as potential biomarkers for the diagnosis of NPSLE. We constructed the diagnostic models with the best value based on these biomarkers for distinguish NPSLE patients from SLE patients and controls. To authenticate the reliability of the 1000-plexed proteins array in our study, we retrieved and verified the expression of some known biomarkers for NPSLE, such as CSF monocyte chemotactic protein-1 level was higher in NPSLE patients [20], CSF interleukin-6 induced intrathecal synthesis of antibodies to aggravate neuronal damage [21]. They were also elevated in our independent Chinese cohort.

TCN2 belongs to the vitamin B12 (VB-12)-binding protein family and can deliver cobalamin to the location of lysosomes released [22]. The decreased concentration of CSF TCN2 in Parkinson’s disease was probably caused by disturbed lysosomal acidification and protease inhibition [23], except that severe deficiency of VB12 was also linked to cognitive decline [24, 25]. According to the ROC analysis, TCN2 owned better diagnostic capabilities (AUC=0.703) than CSF C3 and C4 in NPSLE patients compared with SLE patients. Cystatin E/M (CST6) has been identified mainly as a tumor suppressor protein to regulate legumain activity and metastasis [26, 27]. A member of the cystatin family-cystatin C was useful in evaluating the risk of cardiovascular mortality in SLE patients [28]. In our study, CST6 have been verified the strong correlation with some clinical indexes in NPSLE patients. Thus, the alteration of CST6 in CSF might reflect disease activity or substantial injury. Trappin-2 suppressed the NE-dependent activation of matrix metalloproteinases-9 (MMP-9), to regulate the degradation of the vascular basement membrane during angiogenesis [29]. CSF MMP-9 induced the production of cytokines and leukocyte adhesion molecules by endothelial cells and facilitated the entry of leukocytes and proteins into the CSF [30]. The level of MMP-9 in CSF has been associated with NPSLE in general, and especially cognitive impairment [31]. Even though there was no report between Trappin-2 and NPSLE, we were optimistic that it could be a biomarker associated with MMP-9 to participate in the development of NPSLE. KLK5 is a kallikrein-related peptidase and is recognized as a prognostic biomarker for cancer; more research was urgently needed to discover the role of KLK5 in CSF from NPSLE patients [32, 33]. Baraczka et al. have already measured the increased level of CSF soluble L-selectin in SLE patients with CNS involvement. It was also dramatically correlated with CSF IgG and albumin in our study, which implied the role of L-selectin with presumable BBB disturbances [34].

Besides the five validated proteins, some proteins also showed excellent potential in the involvement of pathological mechanisms in NPSLE patients. As Fig. 2C and Fig. S2C illustrated, VCAM-1 was the key protein with the highest value of BC. Some studies have confirmed that the elevated level of VCAM-1 was positively correlated with the abnormal level of antiphospholipid antibody to aggravate the BBB damage in NPSLE patients [35, 36]. LDLR was the primary receptor for apolipoprotein E (APOE) [37]. A functional interaction between APOE and LDLR influenced regional brain APOE levels [38]. We have reported that the level of APOE was negatively correlated with line orientation scores-one of the indexes of cognition in SLE patients [39]. Meanwhile, the expression of CSF LDLR was increased in NPSLE patients in our study. Thus, it makes sense to discover the function of LDLR-APOE interaction in the molecular mechanisms of NPSLE.

In the course of the literature search for biomarkers, we observed the possible association between some candidate biomarkers with cognitive dysfunction, a most typical symptom of NPSLE, which prompted us to dig into the 1000-plexed proteins array for more information about cognitive dysfunction. RNA-sequencing of the hippocampus from NPSLE model mice has been finished [16]. Hippocampal structural lesions and metabolic abnormalities have been already identified in NPSLE patients and NPSLE model mice [40–42]. Our previous studies have demonstrated the reliability of using lipoproteins and thyroid hormones as biomarkers to distinguish SLE patients with different degrees of cognitive impairment [39]. Hence, we overlapped two subsets of data from the hippocampus in mice and CSF in patients and surprisingly found 6 candidate biomarkers with the same alteration. The expression of these molecules had a strong correlation in two types of samples. The positive correlation of these common molecules from CSF and the hippocampus indicated the relationship between the hippocampus and its immune environment, which might be involved with the pattern and location of these protein expressions.

We firstly applied the 1000-plexed proteins array to screen proteins and discover these biomarkers mentioned above in CSF rather than peripheral blood. Since lupus is a systemic disease, we also confirmed the expression of five biomarkers in the plasma of controls, SLE, and NPSLE patients, using the data in our previous study [43]. There was no dominant variation of these proteins in the plasma among the three groups. Clearly, it was conceivable that our biomarkers played a specific role in the brain of NPSLE patients because of the separation of the peripheral circulatory system and brain. We also compared 29 DEPs to the normal human CSF proteome [44]. Thirteen proteins were not found in normal human CSF, which might be related to leakage due to BBB impairment. It provided a good enlightenment for our subsequent study of the pathogenesis of NPSLE.

In view of previous studies, some studies have already revealed the association of our novel biomarkers and specific brain symptoms mentioned in our study. For example, the plasma level of TCN2 was significantly increased in the newly diagnosed epileptic seizure patients and long-standing grand mal epileptic patients [45]. TCN2 polymorphism was also identified in patients with ischaemic cerebrovascular disease [46]. The serum level of soluble L-selectin was highly increased in ischemic stroke and silent cerebral infarct in children with sickle cell anemia [47, 48]. Although these studies mentioned the serum level of these proteins in brain diseases, they also helped us to discover the function of our CSF biomarkers in NPSLE patients in our study. Nonetheless, due to the diversity of disease manifestations, different races of patients, and limited sample size, we still need more comprehensive research to discover the potential of CSF proteins as biomarkers in NPSLE patients. Likewise, it would be crucial for us to associate the diagnostic biomarkers with specific neuropsychiatric symptoms and elucidate how they are involved in the brain function and pathogenesis of NPSLE.

## Conclusion

Our study focused on the CSF DEPs based on protein array to distinguish NPSLE patients from SLE patients (non-NPSLE) and controls in the Chinese cohort. The novel five biomarkers were applied to construct diagnostic models with sensitivity and specificity for NPSLE patients. We would continue to promote the clinical application of the novel biomarkers in the early clinical diagnosis, treatment, monitoring, and prognosis tracking for NPSLE.

### Supplementary Information

**Additional file 1:** **Fig. S1.** (A). Radar chart showed the expression differences of 17 candidate biomarkers according to the fold change between NPSLE and SLE groups (blue), and between NPSLE and control groups (orange). (B) 17 proteins were selected to construct the PPI network in the STRING database. Different colored lines represented different interactions. **Fig. S2.** (A) PCA was conducted on all DEPs between NPSLE and control groups (upper), and between NPSLE and SLE groups (under). The first two principal components were plotted to show the difference between two groups. (B-C) GO enrichment analysis of 17 candidate biomarkers between NPSLE and control groups (B), and NPSLE and SLE groups (C), including biological process (left) and molecular function (right), arranged by enrichment score. **Fig. S3.** (A) ROC curves using L-selectin (AUC=0.844, *P*=0.0015), Trappin-2 (AUC=0.793, *P*=0.0069) and CST6 (AUC=0.778, *P*=0.00104) for distinguish NPSLE patients from control group. (B) Multiple proteins were combined into panels in distinguishing NPSLE patients from control group, using logistic regression analysis, indicated the highest diagnostic value of combination of L-selectin, Trappin-2 and CST6 with an AUC of 0.884. **Fig. S4.** (A) LASSO regression for screening parameters and mapping each one to a curve between NPSLE patients and controls. (B) Optimal parameter (lambda) selection in the LASSO model via minimum criteria. (C) Prediction factors from diagnostic model for NPSLE determined by logistic regression. (D) ROC analysis of the diagnostic model.

**Additional file 2:** **Table S1.** Demographic and clinical information of the primary cohort for patients used in the 1000-plexed proteins array. **Table S2.** Demographic and clinical information of the secondary cohort for patients used in the 17-plexed customized proteins array. **Table S5.** 29 DEPs (*p*<0.05; FC>1.2 or <0.83) in NPSLE group compared to SLE group, based on array-based screening of 1000 proteins. **Table S6.** Candidate biomarkers were selected from the protein array screening assay. **Table S7.** The diagnostic value of 5 potential biomarkers based on ROC analysis.

**Additional file 3:** **Table S3.** List of proteins identified by 1000-plexed proteins array and raw data.

**Additional file 4:** **Table S4.** List of proteins identified by 17-plexed customized proteins array and raw data.

## Data Availability

The datasets used and/or analyzed in the current study are available from the corresponding author upon reasonable request.

## Abbreviations

ACR: American College of Rheumatology
ADIPOQ: Adiponectin
APOE: Apolipoprotein E
BBB: Blood-brain barrier
BC: Betweenness centrality
BP: Biological process
BST1: Bone marrow stromal cell antigen 1
CC: Cellular component
CCL4: C-C motif chemokine ligand 4
CDH2: N-Cadherin
CNS: Central nervous system
CSF: Cerebrospinal fluid
CST6: Cystatin E/M
CXCL15: Chemokine (C-X-C motif) ligand 15
DEP: Differentially expressed protein
ENG: Endoglin
GDF2: Growth differentiation factor 2
GO: Gene Ontology
IGFBP4: Insulin-like growth factor binding protein 4
KLK5: Kallikrein-related peptidase 5
LAMP1: Lysosomal associated membrane protein 1
LASSO: Least absolute shrinkage and selection operator
LDLR: Low-density lipoprotein receptor
LYM: Lymphocyte
MF: Molecular function
MMP-9: Matrix metalloproteinases-9
NEUT: Neutrophil
NID2: Nidogen 2
NPSLE: Neuropsychiatric systemic lupus erythematosus
PCA: Principal component analysis
PI3: Trappin-2
PTH: Parathyroid hormone
ROC: Receiver operating characteristic curve
SELL: L-selectin
SLE: Systemic lupus erythematosus
SLEDAI: Systemic Lupus Erythematosus Sisease Activity Index
TCN2: Transcobalamin 2
TFF3: Trefoil factor 3
TGF-β3: Transforming growth factor beta 3
THBS2: Thrombospondin 2
TPP1: Tripeptidyl peptidase 1
WBC: White blood cell count
WIF1: WNT inhibitory factor 1
VCAM-1: Vascular cell adhesion molecule 1
