# Supplementary material for: Novel CSF biomarkers for diagnosis and integrated analysis of neuropsychiatric systemic lupus erythematosus: based on antibody profiling
Source: Arthritis Res Ther. 2023 Sep 8;25:165. doi: 10.1186/s13075-023-03146-z (PMC10486090; doi:10.1186/s13075-023-03146-z)

**Supplementary Figures**

**Fig. S1** (A). Radar chart showed the expression differences of 17 candidate biomarkers according to the fold change between NPSLE and SLE groups (blue), and between NPSLE and control groups (orange). (B) 17 proteins were selected to construct the PPI network in the STRING database. Different colored lines represented different interactions.


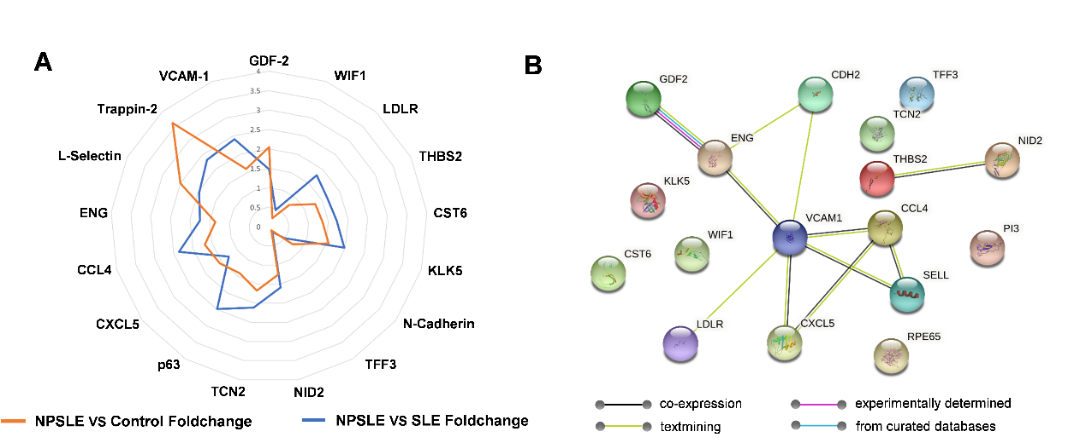


**Fig. S2** (A) PCA was conducted on all DEPs between NPSLE and control groups (upper), and between NPSLE and SLE groups (under). The first two principal components were plotted to show the difference between two groups. (B-C) GO enrichment analysis of 17 candidate biomarkers between NPSLE and control groups (B), and NPSLE and SLE groups (C), including biological process (left) and molecular function (right), arranged by enrichment score.


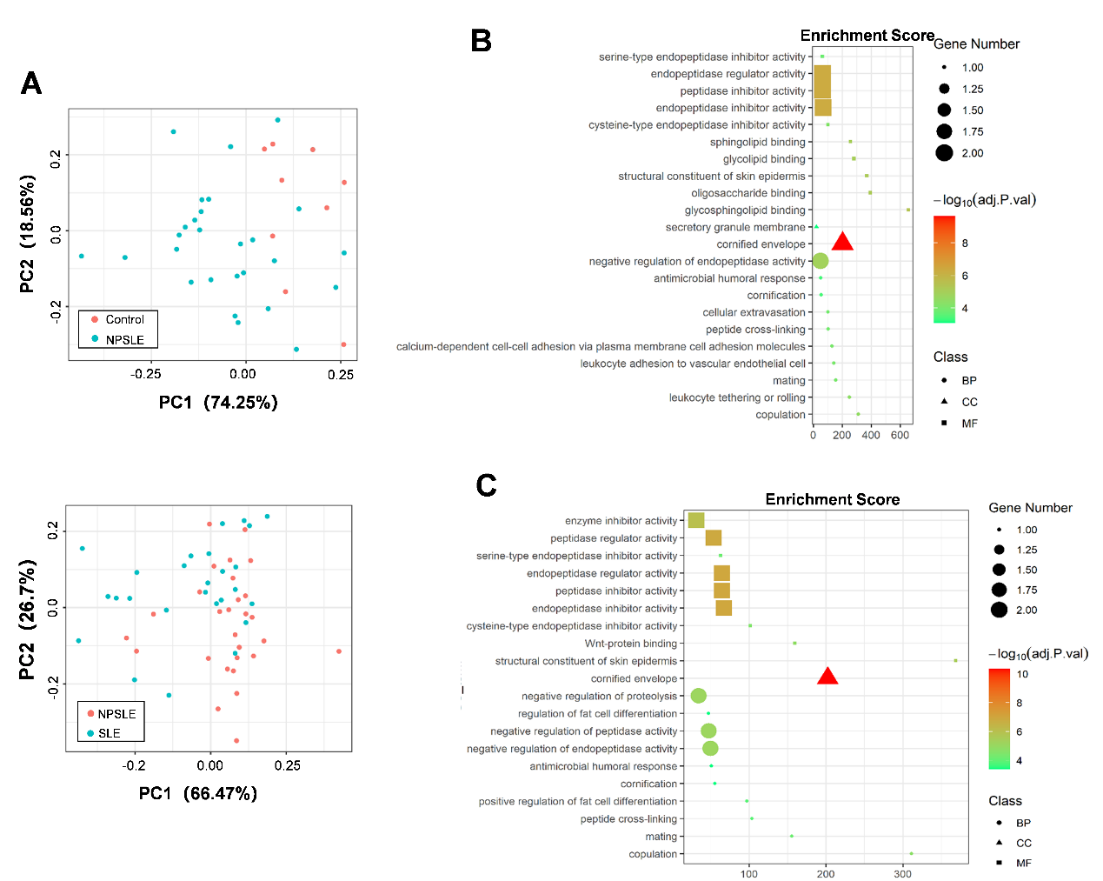


**Fig. S3** (A) ROC curves using L-selectin (AUC=0.844, P=0.0015), Trappin-2 (AUC=0.793, P=0.0069) and CST6 (AUC=0.778, P=0.00104) for distinguish NPSLE patients from control group. (B) Multiple proteins were combined into panels in distinguishing NPSLE patients from control group, using logistic regression analysis, indicated the highest diagnostic value of combination of L-selectin, Trappin-2 and CST6 with an AUC of 0.884.


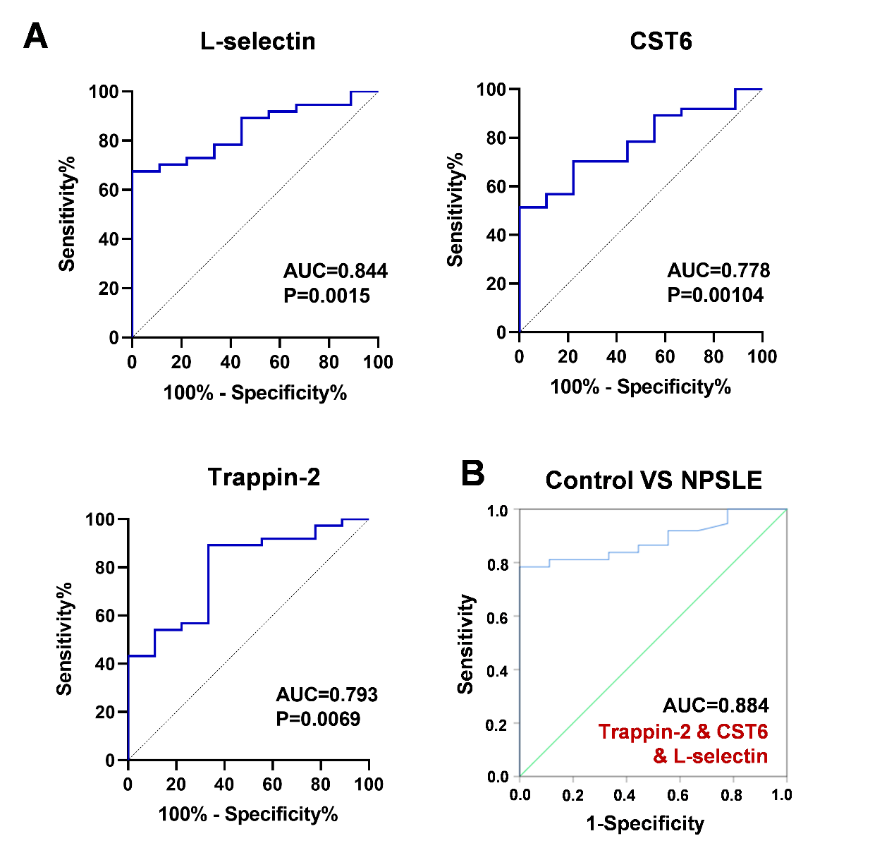


**Fig. S4** (A) LASSO regression for screening parameters and mapping each one to a curve between NPSLE patients and controls. (B) Optimal parameter (lambda) selection in the LASSO model via minimum criteria. (C) Prediction factors from diagnostic model for NPSLE determined by logistic regression. (D) ROC analysis of the diagnostic model.


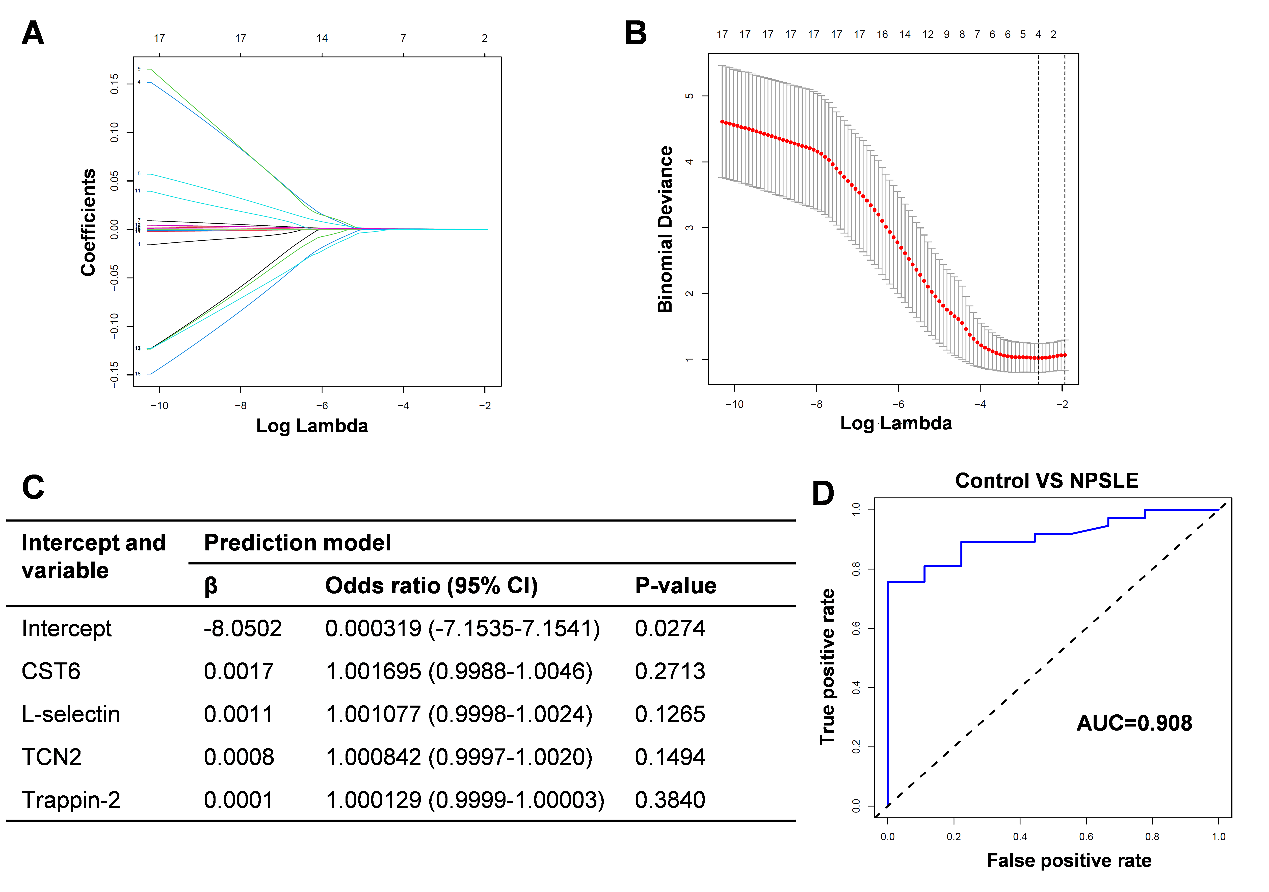

Supplement: Supplementary file 1 — Additional file 1: Fig. S1. (A). Radar chart showed the expression differences of 17 candidate biomarkers according to the fold change between NPSLE and SLE groups (blue), and between NPSLE and control groups (orange). (B) 17 proteins were selected to construct the PPI network in the STRING database. Different colored lines represented different interactions. Fig. S2. (A) PCA was conducted on all DEPs between NPSLE and control groups (upper), and between NPSLE and SLE groups (under). The first two principal components were plotted to show the difference between two groups. (B-C) GO enrichment analysis of 17 candidate biomarkers between NPSLE and control groups (B), and NPSLE and SLE groups (C), including biological process (left) and molecular function (right), arranged by enrichment score. Fig. S3. (A) ROC curves using L-selectin (AUC=0.844, P=0.0015), Trappin-2 (AUC=0.793, P=0.0069) and CST6 (AUC=0.778, P=0.00104) for distinguish NPSLE patients from control group. (B) Multiple proteins were combined into panels in distinguishing NPSLE patients from control group, using logistic regression analysis, indicated the highest diagnostic value of combination of L-selectin, Trappin-2 and CST6 with an AUC of 0.884. Fig. S4. (A) LASSO regression for screening parameters and mapping each one to a curve between NPSLE patients and controls. (B) Optimal parameter (lambda) selection in the LASSO model via minimum criteria. (C) Prediction factors from diagnostic model for NPSLE determined by logistic regression. (D) ROC analysis of the diagnostic model. [file 13075_2023_3146_MOESM1_ESM.docx]
