# Supplementary material for: Novel CSF biomarkers for diagnosis and integrated analysis of neuropsychiatric systemic lupus erythematosus: based on antibody profiling
Source: Arthritis Res Ther. 2023 Sep 8;25:165. doi: 10.1186/s13075-023-03146-z (PMC10486090; doi:10.1186/s13075-023-03146-z)

**Supplementary Tables**

**Table S1.** Demographic and clinical information of the primary cohort for patients used in the 1000-plexed proteins array.

**
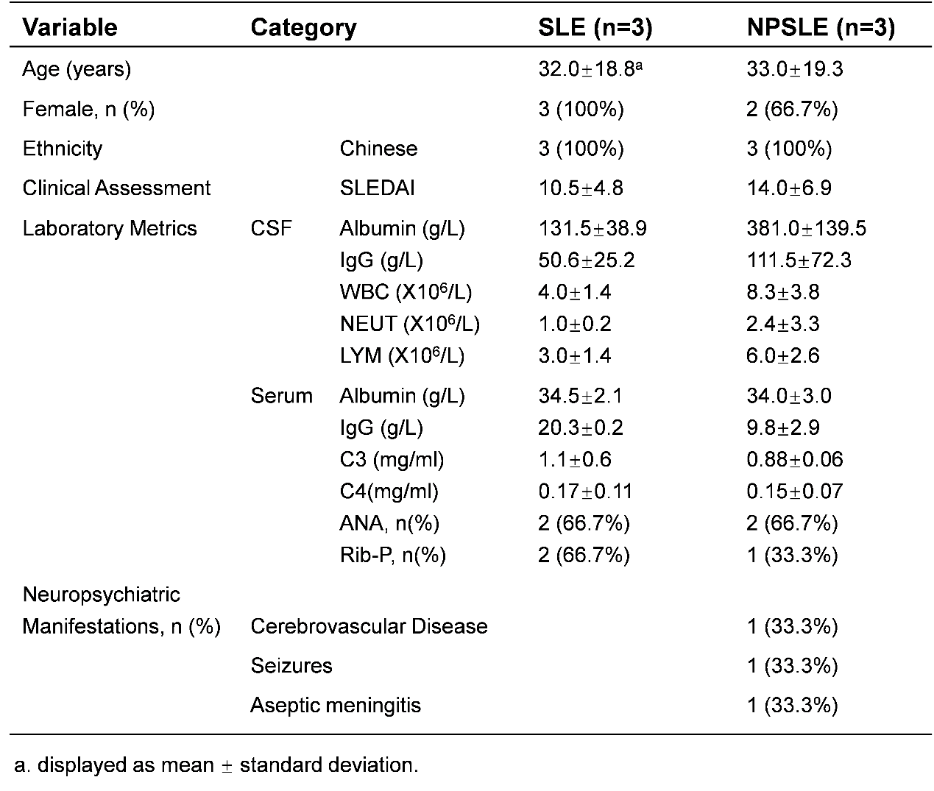
**

**Table S2.** Demographic and clinical information of the secondary cohort for patients used in the 17-plexed customized proteins array.

**
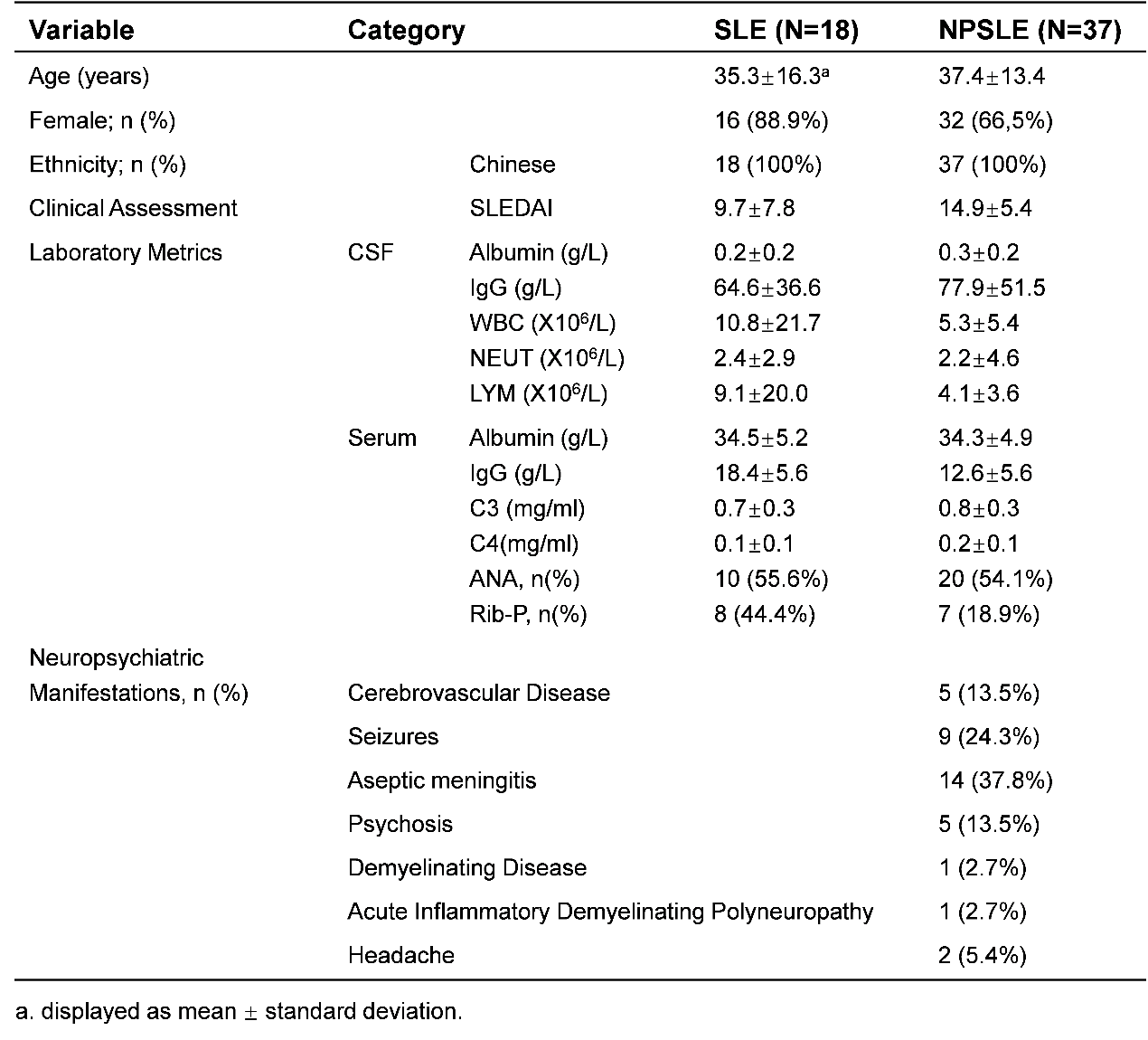
**

**Table S5.** 29 DEPs (p<0.05; FC>1.2 or <0.83) in NPSLE group compared to SLE group, based on array-based screening of 1000 proteins.


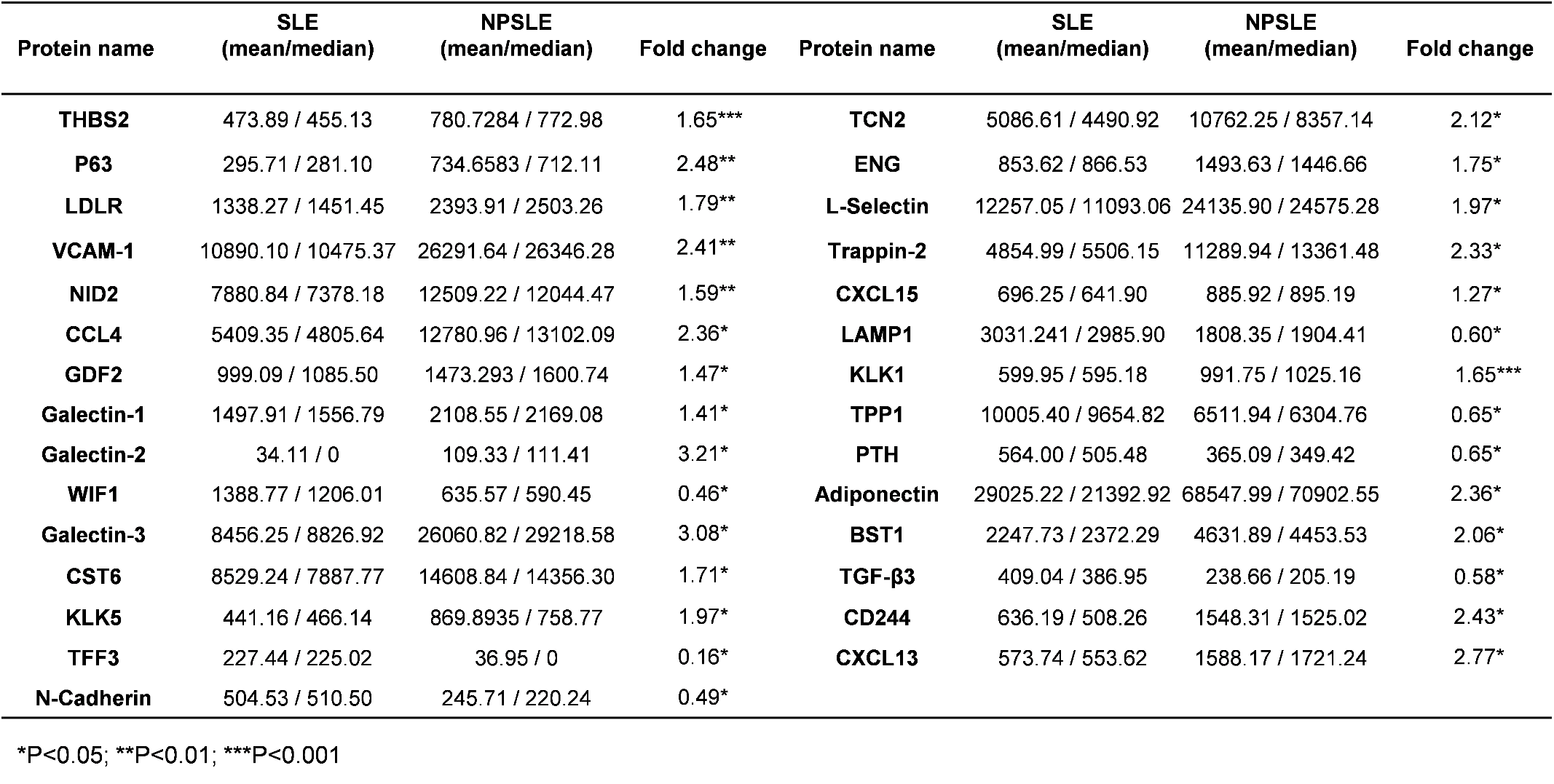


**Table S6.** Candidate biomarkers were selected from the protein array screening assay.


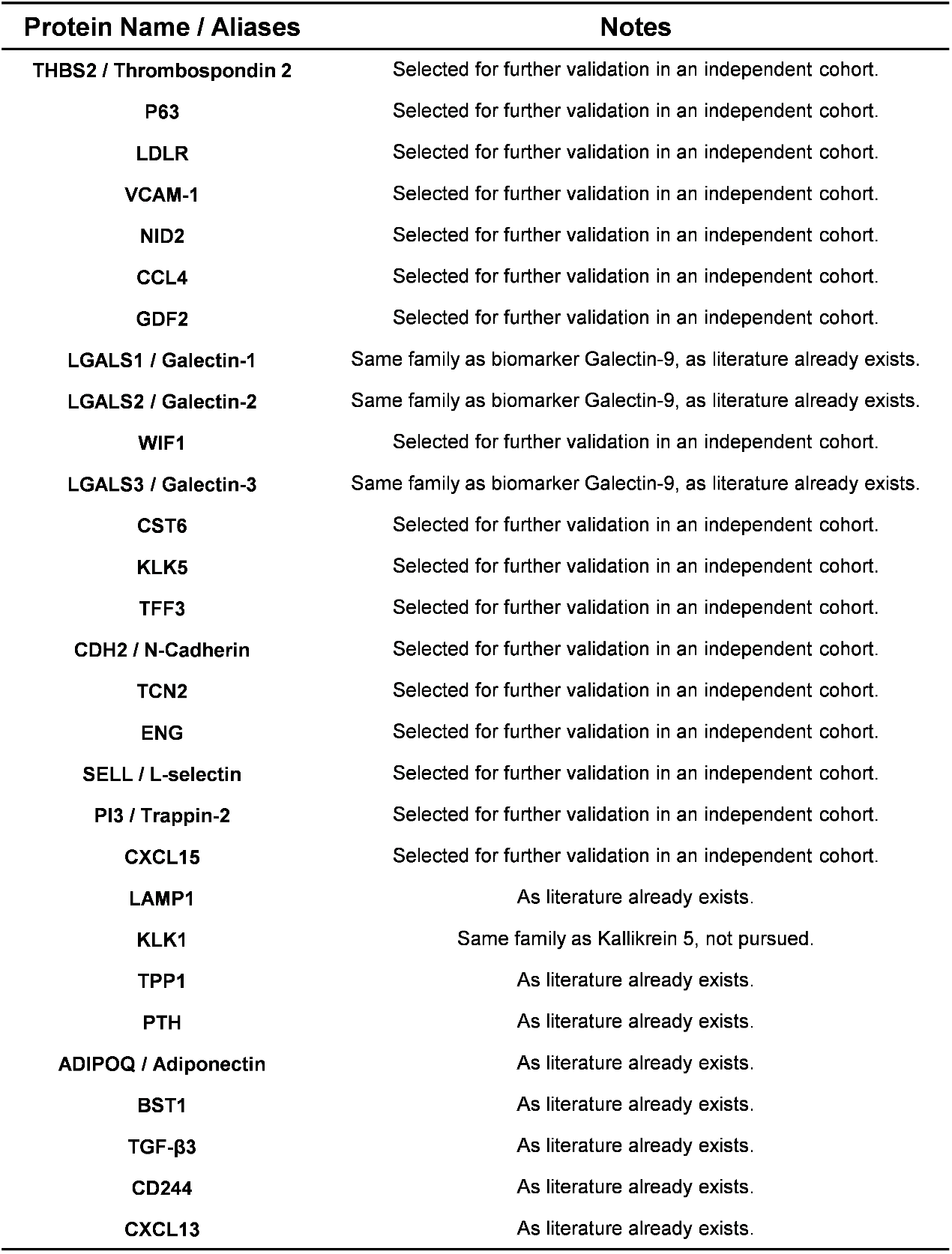


**Table S7.** The diagnostic value of 5 potential biomarkers based on ROC analysis.


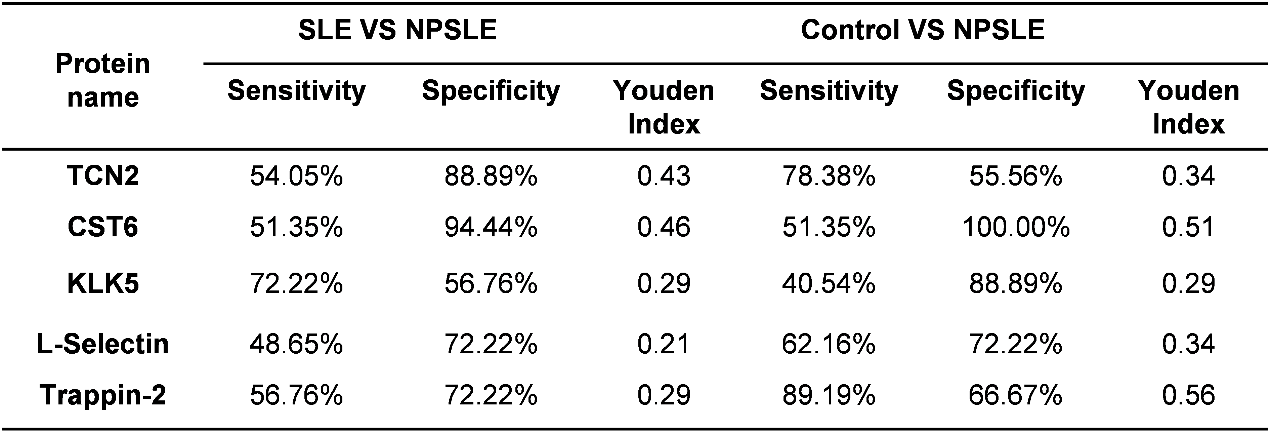

Supplement: Supplementary file 2 — Additional file 2: Table S1. Demographic and clinical information of the primary cohort for patients used in the 1000-plexed proteins array. Table S2. Demographic and clinical information of the secondary cohort for patients used in the 17-plexed customized proteins array. Table S5. 29 DEPs (p<0.05; FC>1.2 or <0.83) in NPSLE group compared to SLE group, based on array-based screening of 1000 proteins. Table S6. Candidate biomarkers were selected from the protein array screening assay. Table S7. The diagnostic value of 5 potential biomarkers based on ROC analysis. [file 13075_2023_3146_MOESM2_ESM.docx]
